# Coping to move again: the psychological dimension of tele-supported rehabilitation after knee arthroplasty—A randomized controlled trial

**DOI:** 10.3389/fdgth.2026.1885446

**Published:** 2026-07-23

**Authors:** Teresa Paolucci, Laura Belinda Rizzo, Alice Cichelli, Federica Bressi, Andrea Bernetti, Giacomo Farì, Marisa Megna, Marco Bonerba, Annalisa Marotta, Marta Di Nicola

**Affiliations:** 1 Department of Medical, Oral and Biotechnological Sciences (DSMOB), “G. d’Annunzio” University of Chieti-Pescara, Chieti, Italy; 2 Center for Sport, Disability and Rehabilitation (CARES), “G. d’Annunzio” University of Chieti-Pescara, Chieti, Italy; 3 Behavioral Imaging and Neural Dynamics Center (BIND), “G. d’Annunzio” University of Chieti-Pescara, Chieti, Italy; 4 “Rehcura” Rehabilitation and Functional Recovery Center, Adelfia, Bari, Italy; 5 Department of Theoretical and Applied Sciences (DiSTA), eCampus University, Novedrate, Como, Italy; 6 Rehabilitation, Fondazione Policlinico Universitario Campus Bio-Medico, Rome, Italy; 7 Department of Medicine and Surgery, Università Campus Bio-Medico, Rome, Italy; 8 Department of Experimental Medicine (Di.Me.S), University of Salento, Lecce, Italy; 9 Infradepartmental University Program of Physical and Rehabilitation Medicine, “V. Fazzi” Hospital, ASL Lecce, Lecce, Italy; 10 Department of Translational Biomedicine and Neuroscience (DiBraiN), University of Bari Aldo Moro, Bari, Italy; 11 Physical and Rehabilitation Medicine Unit, Policlinico University Hospital, Bari, Italy; 12 Laboratory of Biostatistics, Department of Medical, Oral and Biotechnological Sciences, “G. d’ Annunzio” University of Chieti-Pescara, Chieti, Italy; 13 Center for Advanced Studies and Technology (CAST), “G. d’ Annunzio” University of Chieti-Pescara, Chieti, Italy

**Keywords:** activities of daily living, exercise therapy, knee osteoarthritis, pain measurement, patient-reported outcome measures, physical therapy modalities, rehabilitation

## Abstract

**Introduction:**

Persistent pain and functional limitations after total knee arthroplasty (TKA) remain clinical challenges, influenced heavily by psychological factors like coping strategies and treatment expectancy. Tele-rehabilitation offers a promising approach to enhance continuity of care and patient engagement when integrated with psychoeducational support. This study compared the effectiveness of a personalized tele-rehabilitation program incorporating psychoeducational components and caregiver support with conventional outpatient rehabilitation following TKA, exploring the relationships between coping strategies, treatment expectancy, and clinical outcomes.

**Methods:**

This single-blind randomized controlled trial enrolled 48 patients undergoing TKA for knee osteoarthritis, randomly assigned to an experimental group (tele-rehabilitation with psychoeducational support; *n* = 24) or a control group (conventional outpatient rehabilitation; *n* = 24). Forty-four participants completed the study (22 per group) following comparable protocols in frequency and duration. Outcomes—pain (VAS), function (WOMAC, KOOS), autonomy (Barthel Index, Modified Rankin Scale), coping strategies (COPE-NIV), and treatment credibility/expectancy (CEQ)—were assessed at baseline (T0), 4 weeks (T1), and 3 months post-surgery (T2). Non-parametric mixed-effects models and correlation analyses were performed.

**Results:**

Both groups showed significant improvements over time in all clinical outcomes (*p* < 0.001). Significant group time interactions were observed for pain, with the tele-rehabilitation group demonstrating greater reductions in VAS pain (*p* = 0.019), WOMAC Pain (*p* = 0.036), and KOOS Pain (*p* = 0.046). Baseline coping strategies and CEQ scores were comparable between groups. Higher baseline “Social Support” coping was exploratorily associated with greater improvement in selected pain-related outcomes. No significant between-group differences emerged for functional independence or quality of life. No adverse events occurred.

**Discussion:**

A personalized tele-rehabilitation program with psychoeducational components and caregiver support was implemented without adverse events in this sample. No statistically significant between-group differences were observed for functional outcomes, whereas greater reductions in pain were observed in the experimental group. The significant correlation between social support coping and better clinical outcomes suggests the potential importance of a biopsychosocial approach to post-TKA care.

**Clinical Trial Registration:**

ClinicalTrials.gov ID Identifier: NCT07032805.

## 1 Introduction

The number of individuals undergoing total knee arthroplasty (TKA) has grown substantially over recent decades, driven by aging populations, rising incidence of osteoarthritis, and improving surgical techniques and implant designs. TKA is widely regarded as an effective intervention for relieving pain and restoring function in patients with end-stage knee osteoarthritis; nonetheless, a notable proportion of patients continue to experience persistent pain, functional limitations, or reduced satisfaction with outcomes (1). Such observations highlight that successful rehabilitation after TKA involves more than implant mechanics or surgical factors alone: psychological, behavioral, and self-management dimensions play critical roles. Among these, the patient's coping strategies cognitive, emotional, and behavioral responses to pain and rehabilitation demands are emerging as key determinants of rehabilitation success and post-surgical pain experience (2, 3).

Post-operative rehabilitation after TKA aims at restoring range of motion (ROM), improving quadriceps strength, regaining functional mobility (walking, stair climbing, chair rises), and enabling safe return to daily activities. Protocols frequently emphasize early mobilization, goal-oriented physiotherapy, strength training, gait re-education, and home exercise programs. Even with optimal surgical technique and post-operative care, a subset of patients fails to achieve expected functional levels or continue to experience bothersome knee pain beyond the acute phase. A recent systematic review reported persistent knee pain more than three months after TKA in approximately 5%–10% of patients, that is strongly associated with reduced satisfaction and suboptimal functional outcomes. Several factors influence the trajectory of recovery: surgical and implant variables (alignment, component positioning, soft tissue balancing), patient factors (age, comorbidities, preoperative function, BMI), and rehabilitation delivery (timing, intensity, adherence). However, psychological and behavioral dimensions, particularly coping responses, expectations and adherence, are increasingly recognized as significant modulators of outcome (4).

The increasing interest in remote or tele-rehabilitation (home exercise with remote monitoring, teleconsultation, digital feedback) has been prompted by pressures to shorten hospital stays, reduce costs, and support patient self-management (5). Home-based or hybrid rehabilitation models have shown promise but also highlight the importance of engagement and tailored support. The addition of psychoeducational elements—pain education, coping skills training, and motivational interviewing—may help enhance patient engagement, manage expectations, and address fear of movement (kinesiophobia), thus improving both adherence and clinical outcomes (6, 7).

Coping strategies refer to the cognitive and behavioral efforts individuals use to manage internal and external demands that are appraised as taxing or exceeding their resources (8). In surgical recovery, coping encompasses how patients deal with pain, limitations, and adherence to prescribed exercises. In the pain literature, coping strategies are classified as problem-focused vs. emotion-focused, and adaptive (e.g., problem solving, social support) vs. maladaptive (e.g., catastrophizing, denial, avoidance) (9).

In orthopedic patients, coping strategies can influence pain perception, motivation, adherence and ultimately functional recovery. For example, active coping (e.g., self-monitoring progress, adherence to exercises) may lead to better outcomes, while catastrophizing or avoidance can result in poorer functional recovery and persistent pain (3, 10). Psychological factors such as catastrophizing and kinesiophobia have been identified as modifiable risk factors for poor TKA outcomes (2). These findings provide a rationale for integrating coping-enhancing psycho-educational or cognitive-behavioral strategies into rehabilitation.

Evidence linking coping and post-surgical recovery is robust. For instance, in a cohort of athletes following knee surgery, higher pain catastrophizing was associated with poorer return to sport and worse functional recovery, while problem-focused coping predicted improved knee function. In TKA populations, showed that patients with elevated catastrophizing benefited from structured coping skills training, with significant reductions in pain and disability after two months (11).

More recent evidence shows that structured coping-strategy training integrated into occupational therapy improved functional and satisfaction outcomes after TKA (12). The intervention group demonstrated greater gains in the Canadian Occupational Performance Measure (COPM) and reported higher satisfaction. This supports the premise that coping-strategy enhancement, especially when part of a broader rehabilitation plan, can improve outcomes.

Collectively, these studies indicate that maladaptive coping (catastrophizing, avoidance) is associated with persistent pain and functional limitations, while adaptive coping (problem-solving, pacing, self-monitoring) predicts better recovery. Moreover, persistent knee pain after TKA is increasingly conceptualized as a biopsychosocial phenomenon in which central sensitization, fear-avoidance, and coping behavior interplay (13). Therefore, rehabilitation plan by physical and rehabilitation medicine (MFR) that addresses these dimensions may enhance recovery beyond what standard physiotherapy achieves.

Given the role of coping in recovery, optimizing psychological adaptation is essential. Psychoeducational modules, covering pain mechanisms, adaptive coping, and realistic expectations, have been shown to reduce pain catastrophizing and improve outcomes after surgery (6). The use of telemedicine (video sessions, remote monitoring, and mobile applications) can extend these benefits by providing ongoing feedback, remote supervision, and motivational support (5).

Evidence supporting tele-rehabilitation has been reported in both knee osteoarthritis and postoperative arthroplasty populations; however, the available evidence differs with respect to patient characteristics, rehabilitation goals, and clinical outcomes. Therefore, findings from these populations should not be considered interchangeable. Given the growing evidence supporting the role of psychological factors in postoperative recovery, coping strategies may represent clinically relevant variables for understanding inter-individual variability in rehabilitation outcomes. However, whether baseline coping profiles can be used to personalize rehabilitation programs remain uncertain and requires further investigation (14). Multicomponent caregiver-supported tele-rehabilitation programs may therefore improve adherence, functional outcomes, and patient satisfaction, addressing both physical and psychological recovery dimensions (15).

Integrating tele-rehabilitation and psychoeducation thus presents a promising opportunity to address coping strategy modulation as part of comprehensive, personalized rehabilitation after TKA. A program that combines physical exercise, remote feedback, and coping-enhancement education may improve outcomes compared to traditional rehabilitation, which typically emphasizes physical recovery alone.

Based on this rationale, we hypothesize that patients undergoing TKA who participate in a multicomponent caregiver-supported tele-rehabilitation program incorporating remote physiotherapy and standardized psychoeducational support would achieve greater improvements in postoperative pain and functional recovery than patients receiving conventional outpatient rehabilitation. In addition, baseline coping strategies were exploratorily evaluated as potential correlates of rehabilitation outcomes.

The aim is to study the effect and efficacy of two rehabilitation protocols after TKA: one traditional (standard in-person physiotherapy with home exercises) and one enhanced (tele-rehabilitation with psychoeducational components designed to foster adaptive coping and self-management). The study will compare pain, function, adherence, coping adaptation, and satisfaction between groups to evaluate the added value of personalized, tele-rehabilitation after TKA.

## 2 Materials and methods

### 2.1 Study design and ethical considerations

This study was designed as a single-blind, randomized controlled clinical trial conducted in accordance with the Consolidated Standards of Reporting Trials (CONSORT) guidelines (16, 17). The study aimed at evaluating the effect of a tele-rehabilitative intervention following total knee arthroplasty (TKA) for osteoarthritis. The trial was prospectively registered at ClinicalTrials.gov (Identifier: NCT07032805).

All procedures performed in this study were in accordance with the ethical standards of the institutional and/or national research committee and with the 1964 Declaration of Helsinki and its later amendments or comparable ethical standards.

The study protocol was approved by the Ethics Committee **(**Prot. 2114/CEL/2025**)** of the IRCSS Giovanni Paolo II—(Italy), in collaboration with the Department of Oral Medical Sciences and Biotechnologies, “G. d’Annunzio” University of Chieti (Italy). Written informed consent was obtained from all participants prior to enrollment.

### 2.2 Participants, randomization and allocation concealment

**Figure 1:**
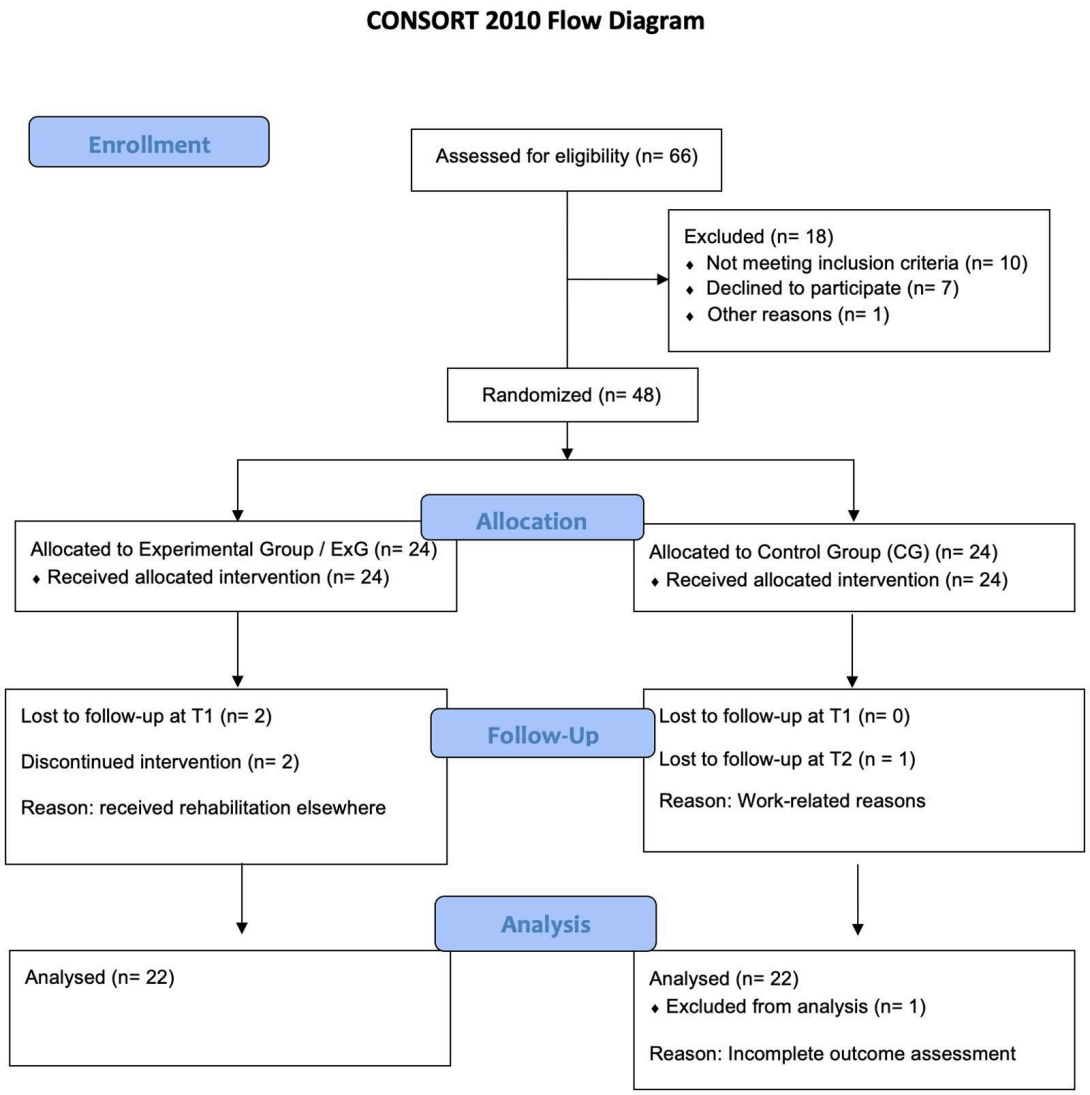
Flowchart.

A total of 48 participants were consecutively recruited among patients referred to postoperative rehabilitation following knee replacement surgery and were randomly allocated to either the experimental group (*n* = 24) or the control group (*n* = 24). Four participants had incomplete follow-up assessments and were therefore not included in the longitudinal outcome analysis; therefore, 44 participants (22 per group) were included in the final analysis (Table 1 and Figure 1). The analysis should therefore be considered a complete-case/completer analysis rather than a formal intention-to-treat analysis.

**Table 1:** Baseline characteristics of the study population.

Variables	Control Group	Experimental Group	Overall	Standardized difference
	*n* = 22	*n* = 22	*n* = 44	
Age (years), median (Q1–Q3)	69.5 (65.2–71.8)	67.5 (64.2–71.0)	68.0 (64.8–71.0)	0.21
Gender, *n* (%)				
Female	17 (77.3)	15 (68.2)	32 (72.7)	0.21
Male	5 (22.7)	7 (31.8)	12 (27.3)	0.21
BMI (kg/m2), median (Q1–Q3)	30.3 (28.4–33.3)	27.4 (26.1–29.3)	28.8 (26.3–32.4)	0.54
Education level, *n* (%)				
Elementary school	6 (27.3)	7 (31.8)	13 (29.5)	0.10
Middle school	9 (40.9)	7 (31.8)	16 (36.4)	0.19
High school	5 (22.7)	7 (31.8)	12 (27.3)	0.21
University degree	2 (9.1)	1 (4.5)	3 (6.8)	0.18
Marital status, *n* (%)				
Married	20 (90.9)	17 (77.3)	37 (84.1)	0.38
Separated	1 (4.5)	0 (0.0)	1 (2.3)	0.31
Single	1 (4.5)	0 (0.0)	1 (2.3)	0.31
Widowed	0 (0.0)	5 (22.7)	5 (11.4)	0.77
Caregiver, *n* (%)				
Yes	14 (63.6)	11 (50.0)	25 (56.8)	0.28
No	8 (36.4)	11 (50.0)	19 (43.2)	0.28
MMSE, median (Q1–Q3)	26.1 (25.0–27.3)	28.3 (26.1–30.0)	26.8 (25.4–28.8)	0.90
VAS pain T0, median (Q1–Q3)	7.0 (5.2–8.0)	7.5 (6.2–10.0)	7.0 (6.0–9.0)	0.59
WOMAC Pain T0, median (Q1–Q3)	11.0 (8.2–12.0)	12.0 (9.2–15.8)	11.5 (8.8–14.0)	0.45
WOMAC Stiffness T0, median (Q1–Q3)	4.0 (3.0–5.0)	5.0 (3.2–6.0)	4.0 (3.0–6.0)	0.61
WOMAC Physical Function T0, median (Q1–Q3)	34.0 (27.5–40.8)	36.0 (20.0–48.0)	35.0 (22.8–44.5)	0.18
WOMAC Total T0, median (Q1–Q3)	50.5 (41.2–55.8)	54.0 (31.2–67.8)	51.5 (31.8–64.5)	0.30
Barthel Index T0, median (Q1–Q3)	85.0 (80.0–85.0)	92.5 (81.2–95.0)	85.0 (80.0–95.0)	0.38
KOOS Symptoms T0, median (Q1–Q3)	54.3 (43.7–66.1)	50.0 (35.7–62.5)	50.0 (38.4–65.2)	0.30
KOOS Pain T0, median (Q1–Q3)	41.7 (34.0–60.4)	37.5 (27.8–54.9)	40.3 (30.6–55.6)	0.44
KOOS ADL T0, median (Q1–Q3)	47.1 (38.6–63.6)	44.1 (29.4–71.0)	45.6 (36.0–67.7)	0.07
KOOS QoL T0, median (Q1-Q3)	31.2 (25.0–43.8)	25.0 (18.8–29.7)	25.0 (18.8–37.5)	0.72
Modified Rankin Scale T0, median (Q1–Q3)	3.0 (3.0–3.0)	3.0 (3.0–4.0)	3.0 (3.0–3.0)	0.53
COPE Social Support T0, median (Q1–Q3)	27.5 (22.0–30.0)	24.0 (22.2–28.8)	25.0 (22.0–30.0)	0.15
COPE Avoidance Strategies T0, median (Q1–Q3)	22.0 (17.8–25.5)	20.0 (18.0–22.8)	21.0 (18.0–23.2)	0.13
COPE Positive Attitude T0, median (Q1–Q3)	27.0 (24.0–30.2)	24.0 (24.0–31.0)	24.0 (24.0–31.2)	0.03
COPE Problem-Oriented Coping T0, median (Q1–Q3)	25.0 (17.0–27.8)	22.5 (18.5–26.8)	23.5 (18.0–27.2)	0.20
COPE Transcendent Orientation T0, median (Q1–Q3)	23.0 (18.5–24.0)	20.0 (18.0–22.0)	22.0 (18.0–24.0)	0.38
CEQ Credibility T0, median (Q1–Q3)	27.0 (24.0–27.0)	24.0 (21.2–24.0)	24.0 (21.8–27.0)	0.51
CEQ Expectancy T0, median (Q1–Q3)	24.5 (22.2–26.0)	24.5 (22.2–25.8)	24.5 (22.0–26.0)	0.02
HADS anxiety at T0, *n* (%)				
Yes	4 (18.2)	6 (27.3)	10 (22.7)	0.22
No	18 (81.8)	16 (72.7)	34 (77.3)	0.22
HADS depression at T0, *n* (%)				
Yes	0 (0.0)	1 (4.5)	1 (2.3)	0.31
No	22 (100.0)	21 (95.5)	43 (97.7)	0.31

The inclusion criteria were: (1) age between 40 and 75 years; (2) primary unilateral total knee arthroplasty performed for end-stage knee osteoarthritis (Kellgren-Lawrence grade III-IV); (3) clinical stability permitting participation in an intensive rehabilitation program; (4) ability to walk independently with or without a walking aid at discharge from inpatient rehabilitation; (5) independence in basic activities of daily living; (6) access to an internet-connected smartphone, tablet, or computer equipped with a camera and microphone; and (7) sufficient digital literacy to participate in videoconference-based rehabilitation sessions independently or with minimal caregiver assistance (“technology compliant”).

The exclusion criteria included revision knee arthroplasty or bilateral procedures, postoperative medical instability or major postoperative complications preventing rehabilitation, the presence of red flags (active malignancy, severe rheumatological disease, uncontrolled systemic disease, or active infection), severe neurological disorders or cognitive impairment interfering with rehabilitation participation, recent myocardial infarction or stroke, thrombocytopenia or bleeding disorders contraindicating intensive exercise, inability to safely participate in home-based rehabilitation because of inadequate technological resources or lack of caregiver support when required, and participation in any additional physiotherapy program during the study period.

Participants were randomized using a simple 1:1 allocation procedure, without block randomization or stratification. The random allocation sequence was generated by an independent researcher not involved in participant recruitment, treatment delivery, or outcome assessment. Allocation concealment was ensured using sealed, opaque, sequentially numbered envelopes, which were opened only after completion of the baseline assessment. Outcome assessors remained blinded to group allocation. Participants in both groups were instructed to avoid any additional physiotherapy or conservative treatments during the study period.

### 2.3 Rehabilitative interventions and adverse events

To avoid bias related to TKA surgery, patients were operated on by three different orthopedic surgeons with at least 5 years of surgical experience who used the same technique and type of prosthesis. Likewise, before starting the rehabilitation program, patients underwent a physical therapy examination by the physical medicine and rehabilitation (PM&R) specialist to access the intensive rehabilitation program and, subsequently, a reassessment before hospital discharge for continuation on an outpatient basis. All enrolled patients were admitted to the intensive rehabilitation program within the first week after surgery, once clinically stable.

For both groups, during the three-week intensive inpatient rehabilitation program, patients underwent two 60-minute physiotherapy sessions and one 30-minute occupational therapy session daily delivered by licensed physiotherapists and occupational therapy experienced in orthopedic rehabilitation.

The physiotherapy program aimed to: postoperative pain and inflammation management, prevention of immobilization complications (deep vein thrombosis, stiffness), progressive restoration of joint mobility (ROM), muscle strengthening (especially quadriceps and gluteal muscles), recovery of walking with assistive devices and progressive independence in daily living activities (ADLs) (18–20).

Patients discharged from post-acute rehabilitation and started on an outpatient rehabilitation program were able to walk either without assistance or with the need for a cane in an outdoor environment. Following discharge, both groups received a rehabilitation program with an equivalent supervised exercise dose. Participants attended two supervised physiotherapy sessions per week, each lasting approximately 60 min, for 12 consecutive weeks (24 supervised sessions; approximately 24 h of supervised rehabilitation in total). The rehabilitation protocol followed the same progression criteria in both groups with respect to range-of-motion exercises, strengthening, gait training, balance exercises, and functional task progression. The primary difference between groups concerned the mode of delivery (tele-rehabilitation vs. conventional outpatient rehabilitation) and the additional psychoeducational and caregiver-supported components included only in the experimental program.

Patients assigned to the experimental group received a preliminary explanation session on how to conduct tele-rehabilitation sessions with a bioengineer and completed a 60-minute session in small groups of 5 people on how to manage the physiotherapy session at home, program progression, and customization based on functional evolution and pain perception in the presence of a physiotherapist, psychologist, and physiatrist.

Table 2 summarizes the progression of the tele-rehabilitation program across the 12-week period.

**Table 2:** 12-Week tele-rehabilitation program (zoom platform).

**Week**	**Session Focus**	**Exercise**	**Sets/Reps**	**Duration/Notes**	**Key Points/Progression**
**1–2**	ROM & Gentle Activation	*Seated knee flexion/extension*	3 × 10–15	10 min	Avoid pain > 4/10 on VAS; use towel roll for support if needed
		*Ankle pumps*	3 × 20	5 min	Prevent DVT; keep toes moving up/down
		*Isometric quad sets*	3 × 10	5 min	Tighten quads, hold 5 s, relax
		*Glute sets/bridges (mini)*	3 × 10	10 min	Lift hips slightly, avoid hyperextension
	Gait & Functional	*Short-distance walking with walker/cane*	10 min	10 min	Focus on heel strike and controlled knee extension
	Cool Down	*Gentle stretching (hamstrings, calves)*	2 × 20 s	5 min	Pain-free stretching
**3–4**	Strength & Balance	*Seated knee extensions with light resistance band*	3 × 10	10 min	Increase resistance gradually
		*Standing mini squats*	3 × 8–10	10 min	Hold back support if needed
		*Heel raises (bilateral, then unilateral)*	3 × 10	5 min	Focus on controlled motion
	Proprioception	*Weight shifting/balance in standing*	3 × 10	5 min	Hold support nearby; eyes open first, then eyes closed if stable
	Gait Training	*Heel-to-toe walking*	2 × 5 min	5–10 min	Improve stride and knee control
	Cool Down	*Gentle hamstring/calf/quad stretch*	2 × 20 s	5 min	Avoid aggressive stretching
**5–6**	Strength & ROM	*Knee extensions with heavier resistance band*	3 × 12	10 min	Increase resistance progressively
		*Step-ups (low step)*	3 × 10	10 min	Start with small height; hold rail/support
		*Side leg raises (lying or standing)*	3 × 12	10 min	Focus on glute medius
	Balance/Proprioception	*Single-leg stance (with support as needed)*	3 × 20–30 s	5 min	Progress to eyes closed
	Functional Mobility	*Sit-to-stand from standard chair*	3 × 10	5 min	Ensure knees fully extend
	Cool Down	*Gentle stretches*	2 × 20 s	5 min	Pain-free
**7–8**	Advanced Strength & Functional	*Knee extension with ankle weights or band*	3 × 12–15	10 min	Focus on controlled motion
		*Step-ups/step-downs (increase height gradually)*	3 × 10	10 min	Add slight tempo increase
		*Mini lunges (assisted/partial)*	3 × 8–10	10 min	Ensure safe knee alignment
	Balance & Coordination	*Single-leg stance on soft surface*	3 × 30 s	5 min	Close eyes if safe; progress as tolerated
	Gait & Function	*Treadmill or walking pattern drills*	10 min	10 min	Focus on stride symmetry
	Cool Down	*Full lower limb stretching*	2 × 20–30 s	5 min	Include quads, hamstrings, calves
**9–10**	Functional Strength	*Wall Squats*	3 × 30–45 s	10 min	Keep back flat against the wall. Knees at 90° max. Progression: Hold weights.
		*Lateral Step-ups*	3 × 12	10 min	Keep pelvis level. Avoid knee valgus during the step up.
	Dynamic Balance	*Tandem walking backwards*	3 × 3 meters	5 min	Toe to heel walking in reverse. Use a wall for safety if needed.
	Endurance/Gait	*Brisk Walking/Treadmill*	15–20 min	15 min	Focus on increasing heart rate slightly. Maintain symmetrical stride length.
	Cool down	*Static Stretching*	2 × 30 s	5 min	Hold position without bouncing. Deep breathing.
**11–12**	Advanced Strength	*Forward Lunges*	3 × 10	10 min	Keep torso upright. Front knee should not pass toes. Progression: No hand support.
		*Power Sit-to-Stand*	3 × 12	5 min	Stand up explosively, sit down slowly. Do not use hands.
	Proprioception	*Single-leg stance with reaching*	3 × 30 s	5 min	Stand on one leg and reach for imaginary objects at different heights/angles.
	Real Life Function	*Reciprocal Stair Climbing*	5–10 min	5–10 min	Foot-over-foot pattern. Focus on controlled descent.
	Cool Down	*Full lower limb relaxation*	3 × 30 s	5 min	Ensure full knee extension and flexion are maintained after exercise.
**Additional rehabilitation principles**	**Description**				
Progression criteria	Progression allowed only if pain ≤ 4/10, correct execution, no compensations				
Target ROM	Extension 0°, progressive flexion >110° where achievable				
Intensity progression	Increase repetitions → resistance → task complexity				
Therapist feedback	Real-time correction via videoconference every session				
Safety monitoring	Pain, swelling, instability, fatigue assessed before each session				

The progression presented in Table 2 was applied throughout the 12-week rehabilitation program. Both groups completed two supervised 60-minute physiotherapy sessions per week (24 session in total). Participants in both groups were additionally instructed to perform the prescribed home exercise program on non-supervised days. Progression was individualized according to pain tolerance (VAS ≤ 4/10), functional performance, and therapist assessment.

During the first week, an additional session on pain self-management was included, held by the psychologist and the physiatrist, also via telemedicine. Sessions were conducted individually with a 1:1 physiotherapist-patient ratio. Sessions were held on a flexible schedule, either in the morning or afternoon, depending on the patient's needs. Rehabilitative program notes: avoid exercises that cause pain above 4/10 on the VAS (21); equipment: resistance bands, ankle weights (optional), chair, step or low stair, stable surface for balance; ensure patient has a safe environment and proper camera positioning. Caregiver availability was recommended for all participants allocated to the tele-rehabilitation program, particularly during the early postoperative period, in order to improve safety, facilitate camera positioning during videoconference sessions, and provide practical assistance when necessary. The level of caregiver involvement was individualized according to each participant's functional status and home environment. Although caregiver support was routinely encouraged throughout the intervention, its intensity and frequency were not prospectively standardized or formally recorded, as it was considered part of the overall rehabilitation context rather than a separate therapeutic intervention. Progression: gradually increase repetitions, resistance, or step height every 1–2 weeks based on tolerance and introduce more challenging balance or functional exercises after week 4. The experimental intervention was intentionally designed as a multicomponent rehabilitation program integrating tele-rehabilitation, psychoeducational support, caregiver involvement, multidisciplinary supervision, and home-based exercise. Therefore, the intervention should be interpreted as a comprehensive biopsychosocial rehabilitation model rather than as tele-rehabilitation alone. Between supervised sessions, participants were instructed to perform the prescribed home exercises on non-supervised days (approximately three additional sessions per week, 20–30 min each). Progression of exercise intensity and complexity was individualized according to pain tolerance (VAS ≤ 4/10 during exercise), functional performance, and therapist clinical judgment during weekly reassessment. Exercise progression was individualized according to each participant's clinical status and functional recovery. Progression was permitted only when the participant completed the prescribed exercises with correct movement quality, without compensatory strategies, and reported pain not exceeding 4/10 on the Visual Analog Scale during or immediately after exercise. If pain exceeded this threshold or persisted for more than 24 h, exercise intensity was reduced by decreasing repetitions, resistance, or task complexity until symptoms stabilized. Target rehabilitation goals included restoration of knee extension to 0° and progressive improvement of knee flexion toward functional ranges (>110° whenever clinically achievable), recovery of independent gait, and progressive restoration of activities of daily living. Resistance was progressed gradually by increasing elastic-band resistance or ankle weights according to individual tolerance, while balance and functional exercises were advanced from stable to more challenging conditions as motor control improved. During supervised tele-rehabilitation sessions, physiotherapists provided real-time visual feedback and verbal corrections regarding exercise execution, posture, movement quality, and breathing strategy. Caregivers assisted when necessary to improve safety and optimize camera positioning during videoconference sessions. Attendance at supervised sessions was recorded for all participants. During each tele-rehabilitation session, therapists also verified completion of the prescribed home exercises and documented any difficulties or adverse symptoms reported by participants. Safety was monitored throughout the intervention. Before each session, participants were asked about pain, swelling, instability, falls, and other adverse symptoms. Sessions were interrupted or modified whenever excessive pain, marked fatigue, dizziness, or other safety concerns emerged. No adverse events requiring medical intervention occurred during the study.

Patients assigned to the control group began an outpatient physiotherapy program that followed the same tele-rehabilitation protocol regarding exercise content, progression criteria, session frequency, and duration, but all supervised sessions were delivered face-to-face in the outpatient setting rather than via tele-rehabilitation. Sessions were performed twice a week, with each session lasting an average of 60 min, for twelve weeks. Participants assigned to the conventional outpatient rehabilitation group followed the same progression criteria and were instructed to perform the same home exercise program between supervised sessions. Therefore, the planned rehabilitation dose was comparable between groups with respect to frequency, duration, and exercise content, while differing in the rehabilitation delivery model and the additional multicomponent support provided to the experimental group.

No adverse events were recorded during both the rehabilitative protocol study.

### 2.4 Outcome measures and assessment timeline

Outcome measures were collected at three time points: *T0* at the beginning of the rehabilitative program, within one week after TKA; *T1* after 4 weeks of rehabilitative treatment; *T2* 3 months after surgery. All assessments were performed by trained evaluators blinded to group allocation. The physical therapy follow-up medical visit for both groups at T2 took place in person.

Pain intensity was assessed using a 10-cm Visual Analog Scale (VAS), ranging from 0 (no pain) to 10 (worst imaginable pain). Participants were asked to rate their current knee pain experienced during routine functional activities at the time of assessment. The VAS was selected as the primary outcome measure because of its sensitivity to changes in postoperative pain following TKA (21).

The WOMAC questionnaire was used to assess knee-related pain, stiffness, and physical function. It consists of 24 items divided into *Pain* (5 items), *Stiffness* (2 items), and *Physical Function/Difficulty* (17 items) subscales, as well as a total score. Scores range from 0 to 96, with higher scores indicating worse pain and disability. The WOMAC has been validated in Italian populations and is widely used in patients undergoing TKA (22, 23).

The KOOS questionnaire evaluates knee-related outcomes across five subscales: *Symptoms, Pain, Activities of Daily Living, Sport and Recreation Function, and Knee-related Quality of Life*. The Sport and Recreation subscale was not included in the analyses. This decision was established *a priori* because the study population consisted predominantly of older adults with a sedentary lifestyle undergoing total knee arthroplasty, whose rehabilitation goals focused on restoring pain-free mobility, independence in activities of daily living, and routine functional activities rather than participation in high-demand sporting or recreational activities. Accordingly, only the KOOS subscales considered clinically relevant to the objectives of the present study (Symptoms, Pain, Activities of Daily Living, and Knee-related Quality of Life) were analyzed. Each subscale is scored from 0 to 100, with higher scores indicating better outcomes. The Italian version has demonstrated good reliability and validity in knee osteoarthritis and postoperative populations (24, 25).

Coping strategies were assessed using the Coping Orientation to Problems Experienced—New Italian Version (COPE-NIV), the validated Italian adaptation of the COPE questionnaire. The instrument consists of 60 items, rated on a 4-point Likert scale (1 = *I usually don't do this at all*; 4 = *I usually do this a lot*). Items are grouped into five higher-order coping dimensions: Social Support, Avoidance Strategies, Positive Attitude, Problem-Oriented Coping, Transcendent Orientation. Each dimension comprises 12 items, yielding a score range from 12 to 48 for each subscale, with higher scores indicating greater use of the corresponding coping strategy (26). For consistency with the original COPE-NVI validation study and to facilitate international readability, the English terminology of the five higher-order coping dimensions (Social Support, Avoidance Strategies, Positive Attitude, Problem-Oriented Coping, and Transcendent Orientation) was used throughout the manuscript and tables.

Cognitive status was evaluated using the Mini-Mental State Examination (MMSE), which assesses orientation, memory, attention, language, and visuospatial skills, with scores ranging from 0 to 30. The MMSE was administered *only at T0* to exclude clinically relevant cognitive impairment (27, 28).

The Credibility/Expectancy Questionnaire (CEQ) was used to assess participants' perceptions of treatment credibility and expectancy (administered after allocation). The CEQ consists of 6 items, divided into two subscales: Credibility, reflecting the perceived logical coherence and plausibility of the treatment and Expectancy, reflecting the anticipated effectiveness of the treatment. Items are rated using Likert-type scales and numerical rating formats. Each subscale yields a score ranging from 3 to 27, with higher scores indicating greater treatment credibility or higher expectancy of benefit, respectively. A total CEQ score can be calculated by summing the two subscale scores, resulting in a total range from 6 to 54 (29).

Global disability was assessed using the Modified Rankin Scale (mRS), which ranges from 0 (no symptoms) to 6 (death). Although originally developed for neurological populations, the mRS was included because it is routinely collected as part of the standardized inpatient and outpatient rehabilitation assessment at our institution. In the present study, it was used exclusively as a complementary measure of overall functional independence and global disability rather than as a knee-specific functional outcome (30).

Functional independence in activities of daily living was assessed using the *Barthel Index (BI)****.*** The Barthel Index evaluates performance in 10 basic activities of daily living, including feeding, bathing, grooming, dressing, bowel and bladder control, toilet use, transfers, mobility, and stair climbing. The total score ranges from *0 to 100*, with higher scores indicating greater independence. Scores are commonly interpreted as follows: *0–20*: total dependency; *21–60*: severe dependency; *61–90*: moderate dependency; *91–99*: slight dependency; *100*: complete independence. The BI is a widely used, reliable, and valid measure of functional disability in rehabilitation settings, including postoperative orthopedic populations (31).

### 2.5 Sample size calculation and statistical analysis

The sample size was calculated using pain intensity measured by the Visual Analog Scale (VAS) as the primary outcome. *A priori* power analysis was performed assuming a statistical power of 90% and a two-tailed alpha level of 0.05. A between-group difference of 2 cm on the VAS was considered clinically meaningful (22), in line with the established minimal clinically important difference (MCID). A standard deviation of 1.5 cm was assumed based on preliminary clinical data from our rehabilitation setting. Based on these assumptions, the analysis indicated that a minimum of 16 participants per group was required. To account for an anticipated dropout rate of approximately 10%, the target sample size was increased to 22 participants per group. The calculation was based on the expected between-group difference in VAS change, rather than on the group × time interaction of the longitudinal model. Therefore, the interaction analyses should be interpreted as supportive longitudinal analyses. The longitudinal analyses were conducted on participants with complete available follow-up data. No imputation of missing outcome data was performed. Therefore, the primary analysis was based on a complete-case approach and did not constitute a formal intention-to-treat analysis including all randomized participants.

Continuous variables were assessed for normality using the Shapiro–Wilk test and found to be non-normally distributed. Consequently, all analyses were performed using non-parametric methods. Continuous data are reported as median and interquartile range (Q1–Q3), while categorical data are presented as absolute frequencies and percentages. Baseline comparisons between experimental and control groups were performed using the Mann–Whitney *U*-test for continuous variables and the *χ*^2^-test or Fisher's exact test, as appropriate, for categorical variables. Longitudinal changes in clinical outcomes (WOMAC, KOOS, VAS, Barthel Index, and Modified Rankin Scale) were analyzed using a rank-based non-parametric model for factorial repeated-measures data, implemented in R with the nparLD package and the f1.ld.f1 function. Group was specified as the between-subject factor, time as the within-subject repeated-measures factor, and the subject identifier was used to account for repeated observations within participants. The model tested the main effects of group and time and the group × time interaction. Because this approach is rank-based, no normality assumption or parametric covariance structure was required. When significant interaction effects were observed, non-parametric *post hoc* comparisons were performed to explore within-group changes over time and between-group differences in change. The analysis was conducted on participants with complete available follow-up data, and no imputation of missing data was performed. Relative variation (Δ) of each outcome was calculated as (T2−T0)/T0 and reported as median and interquartile range. Correlation analyses between relative changes in outcome measures and baseline psychological variables (COPE subscales and CEQ scores) were performed using Spearman's rank correlation coefficient. All tests were two-sided and a *p*-value < 0.05 was considered statistically significant. No formal adjustment for multiple comparisons was applied. Therefore, results concerning secondary outcomes, subscales, and correlation analyses should be interpreted as exploratory and hypothesis-generating, particularly for findings with *p*-values close to the conventional significance threshold. All statistical analyses were conducted using R software (version 4.2; http://www.r-project.org/).

## 3 Results

### 3.1 Baseline characteristics

Of the 48 randomized participants, 44 completed the scheduled follow-up assessments and were included in the complete-case longitudinal analysis; 22 subjects in the control group and 22 in the experimental group. Baseline demographic and clinical characteristics are reported in Table 1.

Overall, participants were predominantly female (72.7%), with a median age of 68.0 years (IQR 64.8–71.0) (see Figure 1).

Changes in WOMAC, KOOS, VAS, Barthel Index (BI), and Modified Rankin Scale (MRS) scores across the three assessment time points (T0, T1, and T2) are summarized in Supplementary Table S1 (in Multimedia Appendix 1). Between-group differences in change from baseline to T2, 95% confidence intervals, and standardized effects sizes were added for the main clinical outcomes. At baseline (T0), no statistically significant differences were observed between experimental and control groups. All outcome measures showed a significant main effect of time (*p* < 0.001), reflecting progressive improvements over the study period in both groups. Significant time × group interactions were found for WOMAC Pain (*p* = 0.036), KOOS Pain (*p* = 0.046), and VAS scores (*p* = 0.019), indicating greater pain reduction in experimental compared with control groups. For all other clinical scores, no significant interactions were detected, suggesting similar temporal trends between the two groups.

To complement *p*-value-based comparisons, absolute changes from baseline, between-group differences in improvement, 95% bootstrap confidence intervals, and Cliff's delta were calculated for the primary and secondary outcomes. The experimental group showed greater improvement in pain-related outcomes, including VAS pain, with a between-group improvement difference of 3.50 points (95% bootstrap CI: 0.00–6.00; Cliff's delta = 0.44), WOMAC Pain, with a difference of 3.50 points (95% bootstrap CI: −0.50 to 7.00; Cliff's delta = 0.43), and KOOS Pain, with a difference of 15.26 points (95% bootstrap CI: −1.38 to 27.50; Cliff's delta = 0.36). For functional outcomes, between-group differences were generally smaller and less consistent. Full details, including median values at each time point and repeated-measures model results, are reported in Supplementary Table S1.

Baseline COPE and Credibility/Expectancy Questionnaire (CEQ) scores are presented in Table 3*.* No statistically significant differences were observed between groups for any COPE subscale or for CEQ credibility and expectancy scores. However, CEQ credibility was numerically higher in the control group and, given the small sample size, this difference should be interpreted cautiously. Overall, coping profiles and treatment expectations appeared broadly comparable at study entry.

**Table 3:** COPE and CEQ scores at baseline (T0) in control and experimental groups.

Variable	**Control Group**	**Experimental Group**	***Mann–Whitney test***
	*n* *=* *22*	*n* *=* *22*	***p-value***
COPE (Social Support)	27.5 (22.0–30.0)	24.0 (22.3–28.8)	*0*.*625*
COPE (Avoidance Strategies)	22.0 (17.8–25.5)	20.0 (18.0–22.8)	*0*.*674*
COPE (Positive Attitude)	27.0 (24.0–30.3)	24.0 (24.0–31.0)	*0*.*930*
COPE (Problem-Oriented Coping)	25.0 (17.0–27.8)	22.5 (18.5–26.8)	*0*.*510*
COPE (Transcendent Orientation)	23.0 (18.5–24.0)	20.0 (18.0–22.0)	*0*.*220*
CEQ (Credibility)	27.0 (24.0–27.0)	24.0 (22.3–26.0)	*0*.*098*
CEQ (Expectancy)	24.5 (22.3–26.0)	24.5 (22.3–25.8)	*0*.*947*

Data are expressed as median and interquartile range (Q1–Q3). For descriptive and comparative purposes, COPE subscale scores can be interpreted as follows: 12–24: low use of the coping strategy; 25–36: moderate use of the coping strategy; 37–48: high use of the coping strategy. No clinical cut-off values are defined for the COPE-NIV; therefore, scores are interpreted dimensionally, allowing for comparisons over time and between groups.

COPE subscale scores were generally within the low-to-moderate range for both groups, while CEQ scores indicated moderate-to-high perceived credibility and expectancy of the intervention.

**Figure 2:**
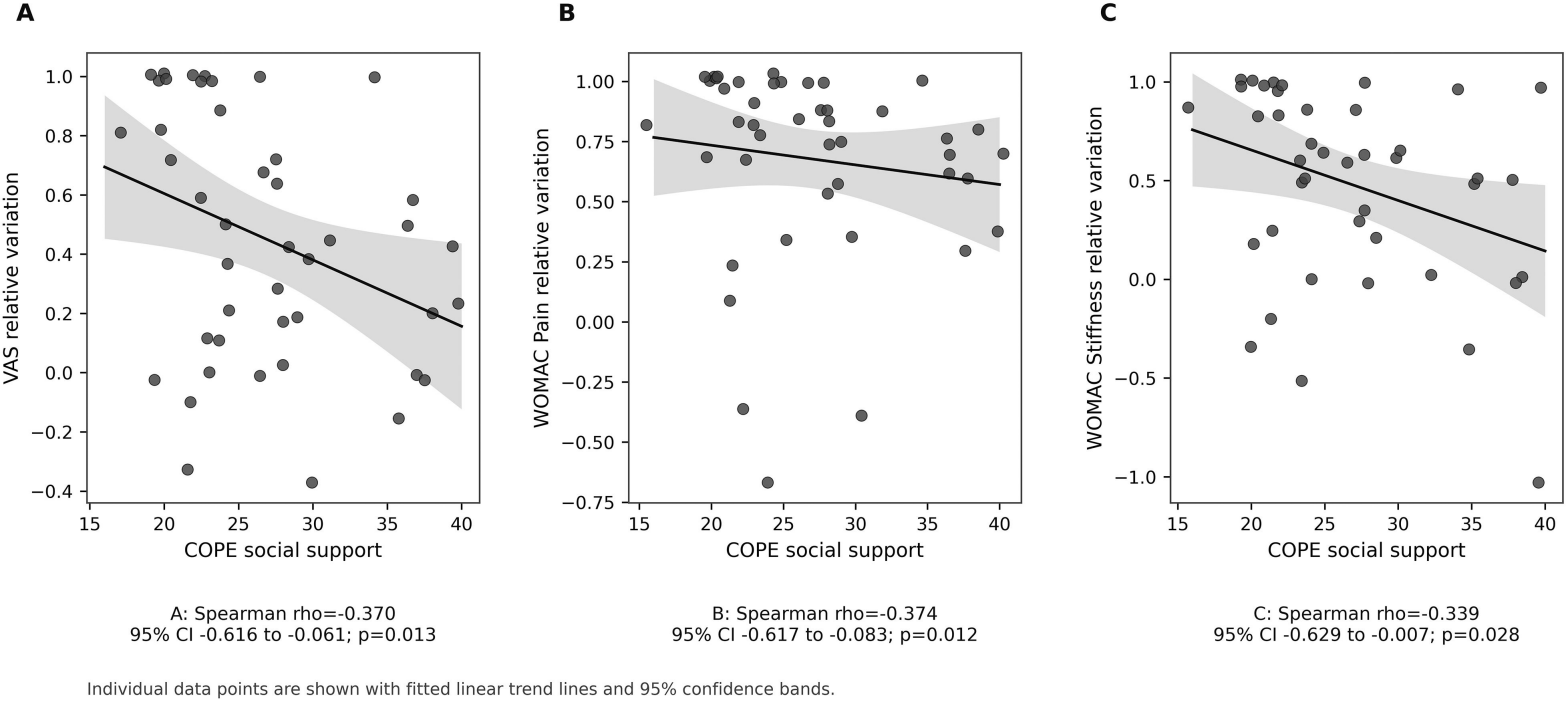
Exploratory associations between baseline COPE social support scores and relative changes in pain-related outcomes. Panels show the associations with VAS pain relative variation **(A)**, WOMAC Pain relative variation **(B)**, and WOMAC Stiffness relative variation **(C)** Individual data points are displayed with fitted trend lines and 95% confidence bands. Spearman's rho, 95% confidence intervals, and *p*-values are reported below each panel. Relative variation was calculated as (T2—T0)/T0.

**Figure 3:**
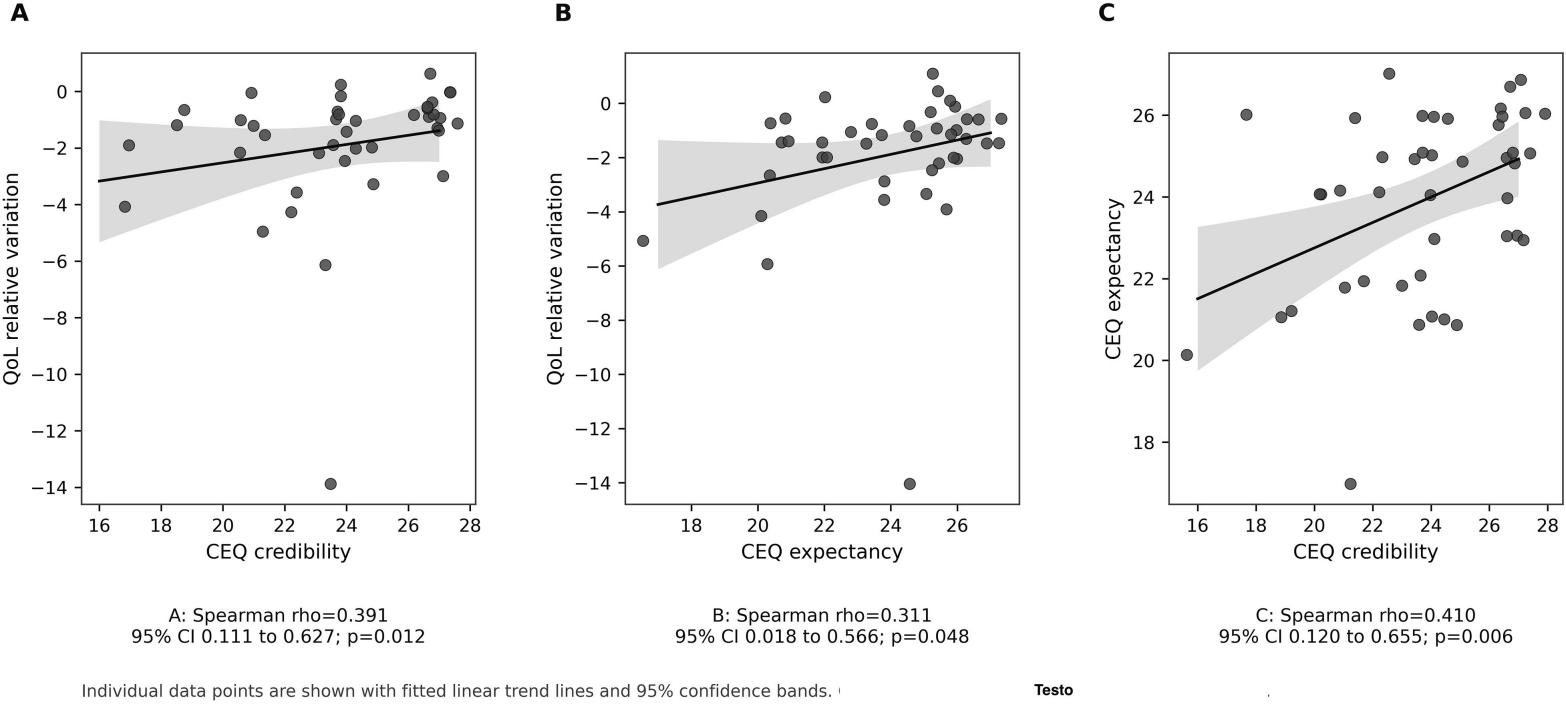
Exploratory associations between CEQ credibility and expectancy scores and KOOS QoL relative variation. Panels show the associations between CEQ credibility and KOOS QoL relative variation **(A)**, CEQ expectancy and QoL relative variation **(B)**, and CEQ credibility and CEQ expectancy **(C)** Individual data points are displayed with fitted trend lines and 95% confidence bands. Spearman's rho, 95% confidence intervals, and p-values are reported below each panel. Legend: For descriptive and comparative purposes, CEQ scores were interpreted as follows: Low credibility/expectancy: 3–11 (subscale)/6–22 (total score); Moderate credibility/expectancy: 12–20 (subscale)/23–40 (total score); High credibility/expectancy: 21–27 (subscale)/41–54 (total score). Higher scores reflect greater perceived plausibility of the intervention and stronger expectations of therapeutic benefit. The CEQ has demonstrated good reliability and validity in clinical and rehabilitation research settings.

Exploratory Spearman correlation analyses between the relative variation (Δ) of clinical outcomes and baseline COPE and CEQ scores are illustrated in Figures 2, 3, respectively. The analyses showed that higher baseline COPE Social Support scores were associated with greater relative improvement in selected pain-related outcomes. Since relative variation was calculated as (T2—T0)/T0, negative coefficients indicate larger reductions over time. Specifically, COPE Social Support was correlated with relative change in VAS pain (rho = −0.370, *p* = 0.013), WOMAC Pain (rho = −0.374, *p* = 0.012), and WOMAC Stiffness (rho = −0.339, *p* = 0.028). These analyses were exploratory and no formal correction for multiple comparisons was applied; therefore, the results should be interpreted as hypothesis-generating.

Additional descriptive correlations were found among pain-related outcome changes: VAS relative variation correlated with WOMAC Pain relative variation (rho = 0.685, 95% CI: 0.459–0.838; *p* < 0.001) and WOMAC Stiffness relative variation (rho = 0.726, 95% CI: 0.515–0.853; *p* < 0.001), while WOMAC Pain and WOMAC Stiffness relative variations were moderately correlated (rho = 0.472, 95% CI: 0.187–0.705; *p* = 0.002).

Regarding adverse events, no adverse events were recorded during both the tele-rehabilitation and outpatient programs. During the rehabilitation protocols, patients did not use additional analgesics other than those usually requested and indicated by the referring physician.

## 4 Discussion

Rehabilitation following total knee arthroplasty (TKA) remains a multifaceted challenge, as postoperative recovery is shaped not only by surgical factors and rehabilitation intensity but also by psychological and contextual determinants, including coping strategies, treatment expectations, and social support. Within this framework, the present study investigated the effects of a personalized rehabilitation pathway integrating tele-rehabilitation and psychoeducational components, with a specific focus on coping strategies, treatment credibility and expectancy, and their relationships with pain and functional outcomes. Importantly, the present study was not designed to isolate the independent effects of tele-rehabilitation itself. Rather, the experimental intervention combined multiple therapeutic components, including tele-rehabilitation, psychoeducational support, caregiver involvement, multidisciplinary supervision, and home-based exercise It should also be emphasized that caregiver involvement formed part of the multicomponent intervention delivered to the experimental group. Consequently, the present study does not allow the independent contribution of caregiver support to be distinguished from that of tele-rehabilitation, psychoeducational sessions, or multidisciplinary supervision. The observed clinical benefits should be interpreted as reflecting the overall effect of this integrated rehabilitation program rather than the specific contribution of tele-rehabilitation alone. The observed clinical effects should therefore be interpreted as resulting from the integrated multicomponent rehabilitation program rather than form any single intervention component.

Evidence regarding coping strategies ad psychoeducational interventions should be considered complementary rather than interchangeable with rehabilitation studies. Whereas rehabilitation trials primarily evaluate physical recovery, psychological studies provide the theoretical rationale for integrating coping-oriented interventions within postoperative rehabilitation programs. The present study was specifically designed to combine these two perspectives within a multicomponent rehabilitation model. In the present cohort, no statistically significant differences were observed between the experimental group (tele-rehabilitation with in-home caregiver support) and the control group (conventional outpatient rehabilitation) with respect to coping strategies assessed by the COPE inventory or treatment credibility and expectancy measured by the CEQ. Both groups exhibited a comparable psychological profile, characterized by moderate use of adaptive coping strategies—such as problem-oriented coping, positive attitude, and social support—and a low reliance on avoidance strategies. This pattern is clinically relevant, as avoidance coping has been consistently associated with poorer functional outcomes, greater pain persistence, and delayed recovery following orthopedic surgery (32). The low use of avoidance strategies observed in both groups suggests a general tendency among patients after TKA to actively engage with rehabilitation demands rather than disengage, which may partly account for the substantial functional improvements observed across groups. These findings are consistent with previous evidence indicating that adaptive coping styles are associated with better rehabilitation adherence and functional recovery after joint replacement (11). Although non-significant, the slightly higher adaptive coping scores observed in the control group may reflect greater exposure to direct therapist–patient interaction in the outpatient setting. However, this did not translate into superior clinical outcomes, suggesting that tele-rehabilitation—when adequately structured and supported—does not compromise psychological engagement with the rehabilitation process.

A relevant exploratory finding of the present study is the negative correlation between baseline social support coping and relative changes in pain-related outcomes. Although greater reliance on social support coping was exploratorily associated with larger improvements in pain-related outcomes, these findings should be interpreted cautiously. Social support coping was assessed only at baseline and was neither experimentally manipulated nor longitudinally monitored. Furthermore, caregiver involvement was encouraged but not standardized or quantitatively recorded, and no mediation analyses were performed. Consequently, the observed associations cannot be interpreted as causal and may instead reflect underlying patient characteristics, family support, motivation, socioeconomic context, or other unmeasured confounding factors. Therefore, these findings should be regarded as hypothesis-generating and warrant confirmation in larger prospective studies specifically designed to investigate the mechanisms linking coping strategies and rehabilitation outcomes. Social support has been shown to enhance rehabilitation adherence, reduce stress responses, and modulate pain perception through both behavioral and neurophysiological mechanisms (33). In orthopedic populations, caregiver involvement and supportive social environments have been associated with improved pain control and functional outcomes following TKA. These findings are particularly relevant in the context of tele-rehabilitation, where the home environment and caregiver presence may amplify the beneficial effects of social support. Overall, the results further support a biopsychosocial model of recovery after joint replacement, in which physical rehabilitation outcomes are closely intertwined with psychological and interpersonal resources (34). Given the number of exploratory correlation analyses performed, these findings should be interpreted cautiously and considered hypothesis-generating.

Regarding treatment-related beliefs, expectancy and credibility scores on the CEQ were comparable between groups, suggesting that differences in outcomes were unlikely to be driven by expectancy or placebo-related effects alone. Nonetheless, a significant positive correlation was observed between CEQ credibility and expectancy, confirming that patients who perceived the rehabilitation program as more credible also reported higher expectations regarding its effectiveness. Moreover, both credibility and expectancy were significantly associated with greater improvements in quality of life. These findings are consistent with previous studies demonstrating that patient expectations and perceived treatment credibility influence patient-reported outcomes after TKA, particularly quality of life and satisfaction. Importantly, the absence of group differences in CEQ scores suggests that the tele-rehabilitation model was perceived as equally credible and acceptable as conventional outpatient rehabilitation, reinforcing its clinical legitimacy and acceptability.

In contrast, evidence derived specifically from patients undergoing total knee arthroplasty provides the most appropriate context for interpreting the present findings. Therefore, comparisons with randomized controlled trials and systematic reviews conducted after arthroplasty are particularly relevant. Both rehabilitation modalities resulted in significant improvements over time across all clinical outcomes, confirming the effectiveness of postoperative rehabilitation irrespective of delivery format. These findings are consistent with a recent systematic review and meta-analysis by Oldrini and colleagues, which compared home-based and conventional rehabilitation following total knee arthroplasty. Although both rehabilitation settings produced significant improvements in pain, function, range of motion, and quality of life, no clinically meaningful differences were observed between approaches, supporting the concept that well-structured tele-rehabilitation may represent an effective alternative to conventional outpatient care. Nevertheless, the authors also emphasized the considerable methodological heterogeneity across studies and the need for further high-quality randomized trials to strengthen the available evidence (35). Baseline comparability between groups further supports the internal validity of these findings. Notably, significant group-by-time interactions were observed for pain-related outcomes (WOMAC Pain, KOOS Pain, and VAS), with the tele-rehabilitation group demonstrating greater pain reduction over time. Recent systematic reviews and meta-analyses have reported that tele-rehabilitation after TKA provides functional outcomes comparable to conventional rehabilitation while offering potential advantages in selected clinical domains, including pain management (36). The home-based context, together with caregiver involvement, may have contributed to the observed pain-related improvements by facilitating engagement with the rehabilitation program; however, these mechanisms were not directly evaluated in the present study. Functional outcomes and global disability measures improved significantly over time in both groups. No statistically significant between-group differences were observed for these outcomes, indicating that both rehabilitation modalities were associated with similar functional improvements during the study period. Accordingly, the present findings indicate comparable functional outcomes between groups within the limits of the statistical analyses performed and should not be interpreted as demonstrating non-inferiority. These results are consistent with randomized controlled trials and cohort studies demonstrating comparable functional recovery and patient satisfaction between tele-rehabilitation and in-person physiotherapy after TKA (37). Evidence derived from patients with knee osteoarthritis should be interpreted separately from studies conducted after total knee arthroplasty because rehabilitation goals, pain mechanisms, and recovery trajectories differ substantially between these populations. Nevertheless, findings from osteoarthritis populations provide an important conceptual framework for understanding the potential role of digitally delivered programs. In this context, our findings are also consistent with the recent systematic review and meta-analysis by Wang et al., which evaluated internet-based telehealth programs for patients with hip and knee osteoarthritis. Across 21 randomized controlled trials, the authors reported moderate-quality evidence supporting improvements in pain, physical function, and self-efficacy compared with conventional care, particularly when exercise-based interventions were incorporated into telehealth programs. Although the population investigated in that review primarily included individuals with osteoarthritis rather than patients following total knee arthroplasty, the results reinforce the growing evidence supporting digitally delivered rehabilitation programs. Our study extends these findings by suggesting that a multicomponent caregiver-supported tele-rehabilitation program may also represent a feasible postoperative rehabilitation strategy following total knee arthroplasty, although these findings should be interpreted cautiously given the exploratory nature of the study (38). The present results are also in agreement with the recent systematic review by Aftab and colleagues, which highlighted that early, structured physiotherapy following total knee arthroplasty is associated with improvements in pain, quality of life, and joint function. Our findings extend this evidence by suggesting that, when delivered through a personalized tele-rehabilitation pathway supported by psychoeducational components and caregiver involvement, these benefits can be achieved while maintaining comparable functional recovery and enhancing pain reduction (20). More recently, additional systematic reviews have continued to support the effectiveness of tele-rehabilitation after TKA, suggesting outcomes comparable to conventional rehabilitation while highlighting the potential added value of digital monitoring and individualized feedback for selected domains such as pain management and patient engagement.

Importantly, coping strategies were not used prospectively to tailor the rehabilitation program. Rather, they were assessed at baseline to characterize participants' psychological profile and to explore their potential association with subsequent clinical outcomes. Consequently, the present findings should not be interpreted as evidence supporting coping-tailored rehabilitation, but rather as exploratory observations that may inform the design of future personalized rehabilitation strategies.

### 4.1 Strengths and limitations

The strengths of this study include its integrative biopsychosocial framework, the simultaneous assessment of psychological variables, clinical outcomes, and rehabilitation modality, and the inclusion of caregiver-supported tele-rehabilitation, which closely reflects real-world rehabilitation practice. The use of validated outcome measures and repeated assessments over time further strengthens the robustness of the findings.

Several limitations should be acknowledged. First, the relatively small sample size limits statistical power and increases uncertainty around some estimates. Moreover, randomization was performed using a simple 1:1 allocation procedure without block randomization or stratification; therefore, clinically relevant baseline imbalance cannot be completely excluded, although standardized differences were reported to better quantify between-group comparability. Second, the analyses were conducted using a complete-case approach rather than a formal intention-to-treat analysis, and no imputation of missing data was performed. Third, no formal adjustment for multiple comparisons was applied; consequently, secondary outcomes, subscale analyses, and correlation analyses should be interpreted as exploratory and hypothesis-generating. The experimental intervention was intentionally designed as a multicomponent rehabilitation program combining tele-rehabilitation, psychoeducational support, individualized remote supervision, and caregiver involvement. Therefore, the independent contribution of each component could not be isolated. Furthermore, caregiver participation and adherence to the home exercise program were encouraged and monitored during supervised sessions but were not prospectively standardized or objectively quantified. Psychological variables, including coping strategies and treatment expectancy, were assessed only at baseline and were not used to tailor the intervention, preventing evaluation of their longitudinal evolution or potential mediating role. In addition, other potentially relevant variables, such as depression, pain catastrophizing, severe obesity, or contralateral lower-limb disorders, were not included as eligibility criteria or adjustment variables and may have influenced postoperative recovery. Finally, the Sport and Recreation subscale of the KOOS questionnaire was not analyzed because of the predominantly sedentary characteristics of the study population, which may reduce comparability with studies reporting the complete KOOS profile. Functional recovery was primarily assessed using patient-reported outcome measures together with the Barthel Index and the Modified Rankin Scale, whereas objective performance-based measures (e.g., Timed Up and Go, 6-minute Walk Test, quadriceps strength, and knee range of motion) were not included. Consequently, the present findings should be interpreted cautiously and should not be considered evidence of causal relationships or of coping-tailored rehabilitation. Future randomized studies with larger samples, longer follow-up, objective adherence monitoring, repeated psychological assessments, and factorial or multi-arm designs are warranted to confirm these findings and to clarify the contribution of the individual intervention components.

### 4.2 Clinical implications and future directions

From a rehabilitative perspective, the present findings suggest that a multicomponent caregiver-supported tele-rehabilitation program may represent a feasible postoperative rehabilitation strategy following TKA, particularly with respect to pain management. However, these findings require confirmation in larger randomized controlled trials before definitive clinical recommendations can be made. Assessment of coping strategies and social support may help identify patients at risk of suboptimal recovery. Future research should investigate targeted psychosocial interventions—such as structured caregiver training, motivational interviewing, and cognitive-behavioral strategies—embedded within tele-rehabilitation pathways and evaluate their long-term clinical and cost-effectiveness.

## 5 Conclusion

This study provides preliminary evidence that a personalized multicomponent caregiver-supported tele-rehabilitation program appeared a feasible alternative to conventional outpatient rehabilitation following total knee arthroplasty. Both rehabilitation modalities were associated with significant improvements in pain, function, disability, and quality of life over time, confirming the overall effectiveness of structured postoperative rehabilitation irrespective of delivery format.

Importantly, no statistically significant between-group differences were observed in functional independence or quality-of-life outcomes, suggesting that both rehabilitation modalities achieved comparable functional recovery. At the same time, the tele-rehabilitation group demonstrated greater improvements in pain-related outcomes. These findings suggest that the multicomponent caregiver-supported tele-rehabilitation program may represent a feasible rehabilitation strategy, although these findings should be interpreted cautiously given the exploratory nature of the study and the relatively small sample size.

From a psychosocial perspective, patients in both groups exhibited comparable coping strategies and treatment-related beliefs, indicating that tele-rehabilitation does not negatively affect psychological engagement or perceived treatment credibility. The observed association between social support coping and greater clinical improvement highlights the relevance of interpersonal and contextual factors in postoperative recovery and is consistent with a biopsychosocial model of rehabilitation after joint replacement.

Taken together, these findings suggest the clinical value of integrating tele-rehabilitation with psychoeducational and caregiver-supported components in post-TKA care pathways. Such models may have the potential to improve accessibility and continuity of care; however, these aspects were not directly evaluated in the present study and therefore require confirmation in future research.

### 5.1 Clinical take-home message

- A multicomponent caregiver-supported tele-rehabilitation appears to be a feasible rehabilitation strategy following total knee arthroplasty.
- In this exploratory study, the multicomponent caregiver-supported tele-rehabilitation program was associated with functional outcomes comparable to conventional outpatient rehabilitation and with greater improvements in pain-related outcomes.
- Baseline social support coping may be associated with greater improvement in pain-related outcomes, although this finding should be considered exploratory.
- Given the exploratory nature of the study and the relatively small sample size, these findings should be interpreted cautiously and confirmed by larger randomized controlled trials before definitive clinical recommendations can be made.

## Data Availability

The raw data supporting the conclusions of this article will be made available by the authors, without undue reservation.
